# Interest in Sexually Transmitted Infections: Analysis of Web Search Data Terms in Eleven Large German Cities from 2015 to 2019

**DOI:** 10.3390/ijerph18052771

**Published:** 2021-03-09

**Authors:** Anna Caroline Pilz, Linda Tizek, Melvin Rüth, Peter Seiringer, Tilo Biedermann, Alexander Zink

**Affiliations:** Department of Dermatology and Allergy, School of Medicine, Technical University of Munich, 80802 Munich, Germany; linda.tizek@tum.de (L.T.); melvin.rueth@gmx.de (M.R.); peter.seiringer@tum.de (P.S.); tilo.biedermann@tum.de (T.B.); alexander.zink@tum.de (A.Z.)

**Keywords:** sexually transmitted diseases, STI, web search data, large german cities

## Abstract

Incidence of sexually transmitted infections (STIs) such as chlamydia, gonorrhea, and syphilis has increased in recent years in the US and in European countries. In order to implement effective educational programs, the interests of target populations have to be identified. Since the internet is an important source of information-gathering on health issues, this study investigates web search data in large German cities related to STIs. Google Ads Keyword Planner was used to identify STI-related terms and their search volume in eleven German cities from June 2015 to May 2019. The data obtained were analyzed descriptively with regard to total search volumes, search volumes of specific thematic areas, and search volumes per 100,000 inhabitants. Overall, 741 terms with a total search volume of 5,142,560 queries were identified, with more than 70% of all search queries including a specific disease and “chlamydia” being the overall most often searched term (*n* = 1,196,160). Time courses of search behavior displayed a continuous interest in STIs with synchronal and national rather than regional peaks. Volumes of search queries lacked periodic patterns. Based on the findings of this study, a more open public discussion about STIs with linkage to increased media coverage and clarification of responsibilities among all STI-treating disciplines concerning management of STIs seem advisable.

## 1. Introduction

Worldwide, more than one million sexually transmitted infections (STIs) occur every day, resulting in approximately 376 million new infections with chlamydia, gonorrhea, syphilis, and trichomoniasis per year [1,2]. Additionally, rising incidences of chlamydia, gonorrhea, and syphilis in the United States (US) and in European countries are present [3,4,5]. In the US, between 2014 and 2018, gonorrhea infections in men increased by over 75% and chlamydia infections by about 38% [3]. In Germany, national data are only available for human immunodeficiency virus (HIV) infections and syphilis. While the incidence of HIV has decreased in the last decade [6], the incidence of syphilis increased by 83% to 9.1 cases/100,000 inhabitants between 2010 and 2017 [7]. In addition to the direct health risks for the affected individual, including imminent and long-term risk such as infertility, STIs present a high socioeconomic burden for healthcare systems. For example, in the United Kingdom, costs for STIs, without the treatment of HIV, were estimated to be GBP 620 million in 2011 [8].

To target STIs effectively, different control strategies are applied worldwide, such as increased screenings combined with partner notifications and simplified therapies in order to reduce the time of infectiousness. Furthermore, sexual health education is intensified [9]. Limited knowledge about STIs in adolescents and underestimation of risks for obtaining STIs in adults emphasize the need for improvement of sexual health education programs [10,11]. One problem regarding STIs is that they are still considered a taboo topic. The perception of STIs is strongly influenced by myths and metaphors, rendering affected persons susceptible to stigmatization and discrimination [12]. Discussions or extraction of information concerning taboo topics such as conversations about suicide or sexually related topics are often transferred to the internet [13,14]. Analyses of web search behavior revealed that the category of sexually related queries is among the most common search categories on the internet [15,16] and that the web is a main source for sexual health information for young people [17].

Health education programs inform target populations and often aim at inducing behavioral changes [18]. In order to be effective, they need to match the interests of their target populations [19,20]. Interviews and surveys are a well-established way to investigate the interest of smaller and specific target populations [21,22]. However, web search data provides information about interests in an almost completely non-selective way. In Germany, about 90% of the population uses the internet [23], with Google being by far (95%) the leading search engine [24]. Up to now, analyses of STI-related web search data focused on correlations of searches for distinct diseases and their infection rates in the context of STI surveillance [25,26]. But search data terms were not yet analyzed in depth and thus it was not yet identified what people want to know about STIs.

In order to improve sexual health education programs, this study investigates German inhabitants’ interest in STIs and analyzes whether there were regional and periodic differences in search interest and frequency in eleven German cities.

## 2. Materials and Methods

### 2.1. Study Design

**Figure 1:**
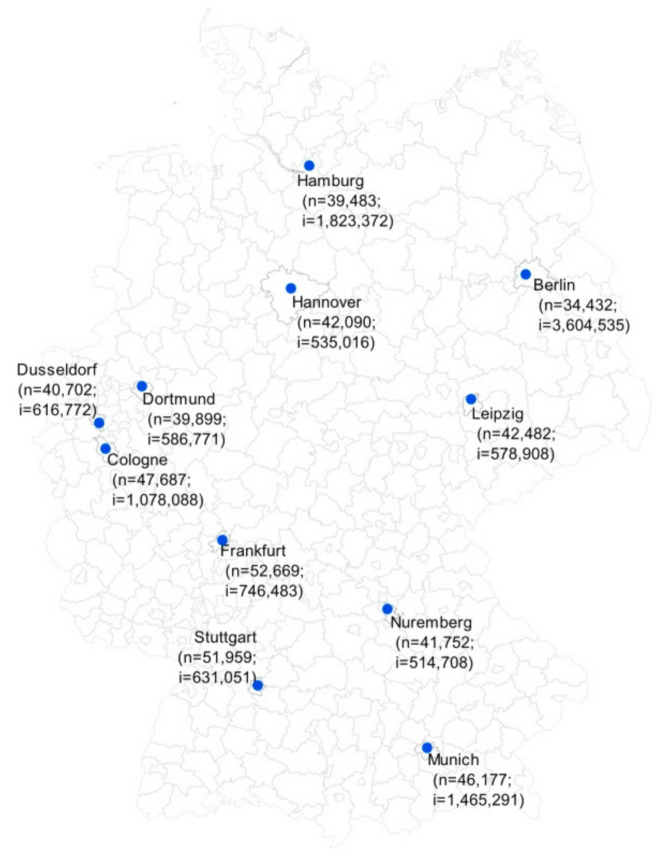
Map showing the regional distribution of the selected eleven German cities (*n*: overall search volume per 100,000 inhabitants; i: number of inhabitants).

A retrospective longitudinal study displaying the web search volume of terms related to STIs in eleven large German cities between June 2015 and May 2019 was conducted. The selected cities were Berlin, Hamburg, Munich, Cologne, Frankfurt, Stuttgart, Dusseldorf, Dortmund, Leipzig, Hannover, and Nuremberg, which are all among the fifteen biggest cities in Germany and are representative of all German regions (Figure 1). By using the Google Ads Keyword Planner, the average monthly search volume of relevant keywords/key terms in German for the term “venereal diseases” was identified. “Venereal diseases” (“Geschlechtskrankheiten”) constitutes the most common German term for STIs. Google Ads Keyword Planner is usually used to optimize placements of advertisements but can also be successfully employed for scientific purposes [27,28]. The search volume indicates the total number of searches for the respective keywords. In this study, search volume was restricted to users whose preferred language was German, and to the area of the selected eleven cities. No institutional review board approval and informed consent was necessary for this study since the data were publicly available.

### 2.2. Categorization

All identified search terms were reviewed, and keywords that did not refer to the initial search term, “venereal diseases” (e.g., disinfection, bacterial diseases list, transmittable diseases), were excluded from further analysis. The remaining keywords were assigned to the following seven categories: “Specific diseases” (e.g., gonorrhea), “gender” (e.g., venereal disease vagina), “symptoms” (e.g., clap symptoms woman), “diagnostics” (e.g., sexually transmitted diseases test), “treatment” (e.g., venereal diseases which physician), “transmission” (e.g., chlamydia infection without intercourse), and “general” (e.g., venereal diseases list). Within the category “gender”, keywords containing male references (e.g., male venereal diseases or venereal diseases penis) and female references (e.g., female venereal diseases or venereal diseases vagina) were differentiated. Keywords that fit into multiple categories were assigned to all of them.

### 2.3. Statistical Analysis

Descriptive data were generated for the identified and categorized keywords. To compare the search volume within the cities, the search volume was calculated in relation to the average number of inhabitants between the years 2015 and 2019 and displayed as number of searches per 100,000 inhabitants [29,30]. To adjust for the proportion of foreigners, who would probably not Google in the German language, data on the average proportion of foreigners between the year 2015 and 2018 were collected [31]. Then, the corresponding number of foreigners in each city was calculated and subtracted from the number of all inhabitants in the respective city. The search volume adjusted to the proportion of foreigners was displayed as the number of searches per 100,000 inhabitants. One-way analysis of variance (ANOVA) was applied to detect differences in search volume per 100,000 inhabitants across the selected cities. 

## 3. Results

### 3.1. Overview

**Figure 2:**
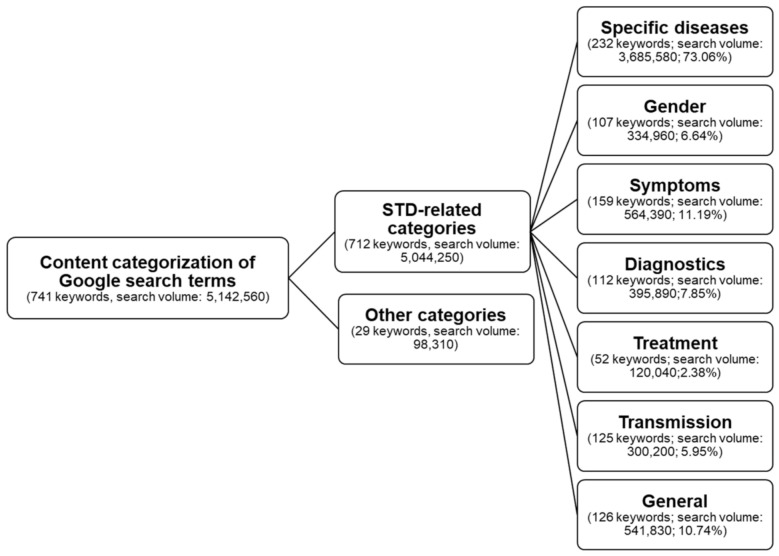
Content categorization of search terms.

A total of 741 keywords associated with the German term for “venereal diseases” were identified. Of these, 29 keywords unrelated to STIs were excluded from further analyses. The remaining 712 keywords with a total search volume of 5,044,250 queries were assigned to seven categories (Figure 2). The highest proportion of search volume referred to the category “specific disease” (73.06%; 232 keywords), followed by “symptoms” (11.19%; 159 keywords) and “general” (10.74%, 126 keywords), whereas only 2.38% (52 keywords) of the search volume was related to “treatment”. Overall, the most frequently searched terms were “chlamydia” (*n* = 1,196,160), “genital warts” (*n* = 559,690), “HIV” (*n* = 458,850), “clap” (*n* = 376,170), and “venereal diseases” (*n* = 268,310) (Supplementary Table S1).

### 3.2. Comparison of Cities

The highest absolute search volume was found in the three largest German cities, Berlin (*n* = 1,241,130), Hamburg (*n* = 719,920), and Munich (*n* = 676,630), while the lowest number of searches was found in Nuremberg (*n* = 214,900). However, when calculating the search volume per 100,000 inhabitants, Frankfurt and Stuttgart had the highest search volumes, with 52,669 and 51,959 queries per 100,000 inhabitants, respectively (Figure 1 and Table 1). This was also the case after the adjustment for the proportion of foreigners in all cities (Frankfurt: 73,834 queries/100,000 inhabitants; Stuttgart; 68,684 queries/100,000 inhabitants). Nevertheless, the differences in the total search volumes per 100,000 inhabitants across cities were not significant.

### 3.3. Most Searched Keywords in Categories

The category “specific diseases” had by far the highest search volume with 3,685,580 queries (30,257 queries/100,000 inhabitants). In this category, the five most common keywords were “chlamydia” (9820 queries/100,000 inhabitants; 32.46%), “genital warts” (4595 queries/100,000 inhabitants; 15.19%), “HIV” (3767 queries/100,000 inhabitants; 12.45%), “clap” (3088 queries/100,000 inhabitants; 10.21%), and “gonorrhea” (1559 queries/100,000 inhabitants; 5.15%). Since clap is the colloquial term for gonorrhea, these two keywords together represent 15.36% of the category “specific diseases”. In the category “symptoms”, the five most common keywords include either “chlamydia” or “clap”. Furthermore, the word “symptoms” itself was predominant as opposed to naming specific symptoms such as “discharge”. In the category “treatment”, four out of the top five keywords contain the word “physician” (21.74%), but none included “chlamydia” (Table 2). The search volume of keywords including male references (*n* = 70, search volume: 209,940 queries) was 1.7 times higher than the search volume of keywords including female references (*n* = 35, search volume: 125,020 queries). Neither the search volume of keywords with female references nor the search volume of keywords with male references per 100,000 inhabitants differed significantly between cities. 

### 3.4. Time Course of Search Queries

Considering all cities, the average monthly number of searches was 9554, resulting in 863 queries per 100,000 inhabitants. The highest number of searches was in October 2015 (*n* = 131,660; 1081 searches/100,000 inhabitants) and the lowest in August 2016 (*n* = 88,480; 726 searches/100,000 inhabitants). The biggest range in number of searches within one city was found in Dusseldorf with 1678 searches per 100,000 inhabitants in June 2015 compared to 694 searches per 100,000 inhabitants in April 2016 and August 2018, displaying a range of 984. 

During the whole study period, no seasonal variations were detected, but a somewhat parallel search behavior between the cities was recognized. Eight cities had the highest or second highest number of searches in October 2015. The other cities were Cologne (July 2017; 1242 searches/100,000 inhabitants), Frankfurt (November 2017; 1302 searches/100,000 inhabitants), Dusseldorf (June 2015; 1678 searches/100,000 inhabitants), and Leipzig (January 2019; 1083 searches/100,000 inhabitants). Another common peak was identified in November 2017.

**Figure 3:**
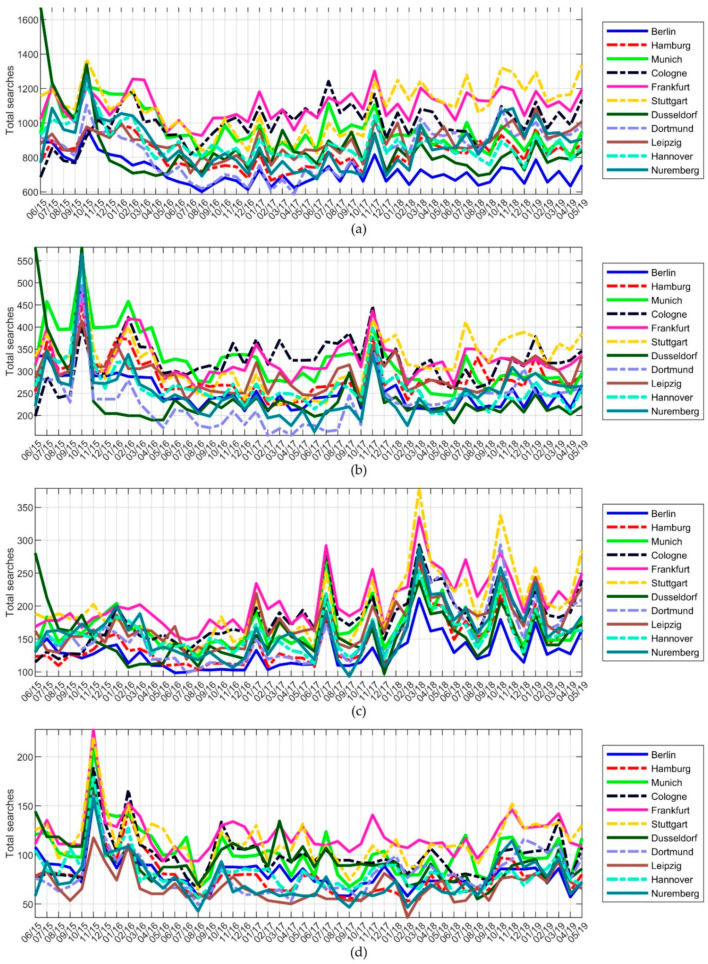
Trends in Google search volumes from June 2015 to May 2019 per 100,000 inhabitants. Sum of all search terms (**a**) related to STIs, (**b**) containing “chlamydia”, (**c**) containing “gonorrhea/clap”, and (**d**) containing “HIV”.

In addition, the time course of search terms related to chlamydia, gonorrhea/clap, and HIV was investigated. Regarding all keywords containing “chlamydia”, two main peaks were observed in October 2015 and November 2017. Keywords with “gonorrhea/clap” showed especially high peaks in July 2017, in March 2018, and in October 2018, and keywords containing “HIV” peaked in November 2015 and February 2016 (Figure 3). 

## 4. Discussion

The aim of the presented study was to assess STI-related web search data to examine people’s interest and to assess whether there were regional or periodic differences across German cities. It was found that the vast majority of searches focused on specific diseases such as chlamydia and gonorrhea. No considerable differences between the cities regarding search interest and time course were observed.

During the analyzed time period of four years, a total of 5,142,560 queries were identified in the eleven large German cities examined, indicating 41,411 queries per 100,000 inhabitants. A trend toward fewer searches per 100,000 inhabitants in larger cities compared to smaller cities was seen. This observation remained after adjustment to the proportion of foreigners in the analyzed cities and has already been described in the context of other Google data analyses [32,33]. Overall, the number of searches per 100,000 inhabitants related to STIs was nearly twice as high than that of “pruritus” (*n*= 21,701 searches/100,000 inhabitants) and also higher than that of “skin cancer” (*n* = 35,573 searches/100,000 inhabitants) [32,33], representing highly prevalent disease conditions. Chronic pruritus affects approximately 14% of the general population in Germany at any time, and skin cancer represents the most common malignancy in Germany [34,35]. With regard to skin cancer, one possible explanation might be the age difference. Skin cancer mainly appears in the elderly, who use the internet less often [23]. A second reason for the higher number of searches regarding STIs, which show lower estimated prevalences than pruritus and skin cancer, might be that STIs are stigmatized and to some extent still taboo [12,36]. Therefore, STIs can be regarded as a highly relevant online search topic. As a consequence, public health institutions should try to discuss STIs more openly. One possibility would be to offer interactive seminars in schools which combine knowledge transfer and reduction of insecurities about STIs.

Analyzing the categories and their most frequent keywords, it immediately becomes apparent that more than 70% of all searches included a specific disease, especially chlamydia. Chlamydia represents the most common bacterial STI in Germany [37]. Additionally, annual screening is offered to women under 25 years and every pregnant woman, and every woman undergoing an abortion is tested for chlamydia [38]. Therefore, many women will hear about chlamydia for the first time in the setting of a screening and may look it up online afterwards. The described factors might be reasons “chlamydia” is the most Googled STI in Germany. Moreover, the search term “chlamydia” was present in the top 5 search terms of all categories except for “treatment” and “general”, though in the latter, it was excluded by default. In Germany, women with suspected STIs are usually treated by gynecologists, especially with regard to chlamydia. However, for men or people at high risk who require regular screenings for all STIs, it is often not evident which physician is the primary contact person. Dermatologists, urologists, and general practitioners share the medical care of these patients [39]. It seems that there is a great need to further clarify responsibilities among medical disciplines especially for the treatment of men since the category “treatment” contains search terms including “physician” and “clap” rather than “chlamydia” and since the number of search terms including “men” was 1.7 times more frequent than those including “women”. Hence, a public health measure could be the creation of official web sites that list detailed information about the availability and the range of services of all public health and medical institutions involved in the care of STIs.

A relatively consistent monthly search volume was seen during the whole study period except for a few peaks. Nevertheless, the majority of cities had their largest search volume in October 2015, which was mainly caused by an increase of searches for chlamydia-related keywords, and which indicates a national rather than regional trend. In contrast to studies that investigated pruritus, skin cancer, or borreliosis, no seasonal variations were detected [28,32,33], which suggests that interest in STIs is independent of periodic external factors such as climate. The few observed national peaks in search volume may be due to increased media coverage, as seen in other studies [40,41]. A recent study showed that Google Trend data in relation to COVID-19 were rather associated with media coverage than epidemiologic data [42]. In the presented study, for example, a high increase in the national search volume for “gonorrhea” was observed in July 2017 when the World Health Organization warned against antibiotic resistances to gonorrhea [43] and in March 2018, when the first case of this multiresistant germ was published in Britain [44]. Both incidents were intensively featured in the main German newspapers and television news [45,46,47]. Some peaks might be due to the coverage in popular German television series, which are watched by approximately 650,000 to 800,000 people every day, as in October 2018 or November 2017, when a protagonist got infected with gonorrhea [48] or chlamydia, respectively. [49]. Concerning HIV, peaks in search volume were seen in November 2015 and February 2016. In November 2015, Charlie Sheen, an actor of world renown, revealed his HIV infection on NBC’s *Today* show, and in February 2016, German scientists reported a breakthrough toward curing HIV [50,51]. Therefore, with regard to the optimization of educational programs, the timing of campaigns should be carefully chosen. For example, information on STIs could appear in commercial breaks of episodes of TV series in which STIs are addressed.

In general, the internet is frequently used as a source for health-related information [16,52], and search engine data have been successfully used in prior studies to describe population interests and behavior with regard to public health topics [27,28,53,54]. The advantage is the great amount of data, which can be easily and anonymously acquired from millions of people. By analyzing the search terms, it is possible to get an overview of people’s interest and thereby reveal unmet needs that are not seen in everyday practice. This is especially true for topics which are experienced as shameful, such as STIs. In contrast to the alternative of, e.g., (online) questionnaires, analyses of search engine data display hypothesis-free approaches. Additionally, there are no wordings, formats or contexts of questions that can influence the population studied, and participation biases are eliminated [55,56]. Conversely, search term data do not include any demographic information, which renders it hard to identify interests of subgroups. Furthermore, the presented data might not be fully representative of the general population as internet users tend to be younger [57]. Moreover, in this study, only data from users whose preferred language was German and who lived in the area of the selected eleven cities were analyzed. Therefore, no statement about rural areas and non-German speaking communities was possible. However, STIs affect primarily younger people, which may attenuate the effect of this limitation [5,58]. Another limitation is that Google presents an automatic completion of search terms, which could influence people’s search queries. Google Ads Keyword planner also only displays estimated, not exact, search volumes, and related keywords/key terms are automatically provided by an unknown algorithm. Since no general reporting requirement for chlamydia and/or gonorrhea exists in Germany, no nationwide epidemiological data are available for these diseases. Although this would be very helpful for public health matters in general, it appears unlikely that positive correlations to disease incidences could have been made in this study. The observed uniform national monthly trends shown are at least partly explained by enhanced public attention following increased media coverage of news or storylines in television series. Furthermore, with regard to chlamydia, the most often searched-for keyword, testing is often done in screening scenarios, which are independent of the time of infection.

## 5. Conclusions

In summary, the study results showed a relatively high search volume related to STIs in German cities, with search queries focusing on specific diseases, especially chlamydia and gonorrhea. Furthermore, instead of regional peaks, national trends with no seasonal correlations were seen. The knowledge gained may be helpful for the planning of big educational campaigns as well as future awareness and prevention strategies. It may be advisable to intensify efforts to discuss STIs more publicly, to clarify responsibilities among STI-treating disciplines especially for management of men with STIs, and to link educational programs directly to broadcast news when public attention is already present.

## Figures and Tables

**Table 1:** Number of searches per 100,000 inhabitants according to categories of sexually transmitted infection (STI)-related keywords in 11 German cities from June 2015 to May 2019.

City	Avg. Number of Inhabitants 2015–2019, (Overall Search Volume) ^1^	Avg. Proportion of Foreigners 2015–2018 ^2^ (%), (Adjusted Overall Search Volume) ^1^	Categories and Number of Searches/100,000 Inhabitants, *n* (%) ^3^						
			Specific Diseases (k = 232) ^4^	Gender (k = 107)	Symptoms (k = 159)	Diagnostics (k = 112)	Treatment (k = 52)	Transmission (k = 125)	General (k = 126)
Berlin	3,604,535	17.08	26,626	1969	3620	2393	691	1559	3505
	(*n* = 34,432)	(*n* = 41,522)	(77.33)	(5.72)	(10.51)	(6.95)	(2.01)	(4.53)	(10.18)
Hamburg	1,823,372	15.73	29,324	2437	4330	3056	846	2057	4162
	(*n* = 39,483)	(*n* = 46,850)	(74.27)	(6.17)	(10.97)	(7.74)	(2.14)	(5.21)	(10.54)
Munich	1,465,291	25.65	34,178	2809	4939	3754	1005	2537	4840
	(*n* = 46,177)	(*n* = 62,108)	(74.02)	(6.08)	(10.70)	(8.13)	(2.18)	(5.49)	(10.48)
Cologne	1,078,088	19	34,891	3258	5538	3631	1170	2947	5010
	(*n* = 47,687)	(*n* = 58,873)	(73.17)	(6.83)	(11.61)	(7.61)	(2.45)	(6.18)	(10.51)
Frankfurt	746,483	28.63	37,351	3629	6090	4442	1373	3555	5850
	(*n* = 52,669)	(*n* = 73,834)	(70.88)	(6.89)	(11.56)	(8.43)	(2.61)	(6.75)	(11.10)
Stuttgart	631,051	24.36	36,157	3786	6126	4534	1342	3689	5832
	(*n* = 51,959)	(*n* = 68,684)	(69.59)	(7.29)	(11.79)	(8.73)	(2.58)	(7.10)	(11.22)
Dusseldorf	616,772	19.45	28,378	3011	4770	3449	1161	2935	4538
	(*n* = 40,702)	(*n* = 50,530)	(69.72)	(7.40)	(11.72)	(8.47)	(2.85)	(7.21)	(11.15)
Dortmund	586,771	16.65	27,372	3279	4799	3047	1111	2930	4705
	(*n* = 39,899)	(*n* = 47,870)	(68.60)	(8.22)	(12.03)	(7.64)	(2.78)	(7.34)	(11.79)
Leipzig	578,908	8.53	29,626	3408	5032	3493	1168	3244	4901
	(*n* = 42,482)	(*n* = 46,441)	(69.74)	(8.02)	(11.84)	(8.22)	(2.75)	(7.64)	(11.54)
Hannover	535,016	21.48	29,306	3196	4807	3602	1256	3034	4594
	(*n* = 42,090)	(*n* = 50,513)	(69.63)	(7.59)	(11.42)	(8.56)	(2.98)	(7.21)	(10.92)
Nuremberg	514,708	16.68	27,831	3425	5119	3757	1259	3388	4985
	(*n* = 41,752)	(*n* = 53,170)	(66.66)	(8.20)	(12.26)	(9.00)	(3.02)	(8.12)	(11.94)
All cities	12,180,995	19.02	30,257	2750	4633	3250	985	2465	4448
	(*n* = 41,411)	(*n* = 51,138)	(73.06)	(6.64)	(11.19)	(7.85)	(2.38)	(5.95)	(10.74)

^1^ Overall search volume/100,000 inhabitants. ^2^ Data were not yet available for 2019 at the time of analysis (November 2019). ^3^ The cumulative percentage might be over 100% since keywords could have been attributed to multiple categories. ^4^ Number of keywords.

**Table 2:** The five most-searched-for terms within each category across all examined cities expressed as search queries per 100,000 inhabitants.

Category and Search Terms	*n*^1^ (%)
**Specific diseases (*n* = 30,257)**	
Chlamydia	9820 (32.46)
Genital warts	4595 (15.19)
HIV	3767 (12.45)
Clap	3088 (10.21)
Gonorrhea	1559 (5.15)
**Gender (*n* = 2750)**	
Chlamydia symptoms woman	294 (10.69)
burning urethra man venereal disease	199 (7.22)
incubation period chlamydia man	165 (6.00)
bacterial infection genital area man	131 (4.75)
venereal disease vagina	128 (4.66)
**Symptoms (*n* = 4633)**	
Chlamydia symptoms	909 (19.62)
Clap symptoms	403 (8.70)
Chlamydia symptoms woman	294 (6.34)
Chlamydia symptoms man	199 (4.29)
Clap symptoms woman	131 (2.82)
**Diagnostics (*n* = 3250)**	
Chlamydia test	808 (24.85)
Venereal diseases test	382 (11.75)
Test venereal diseases	111 (3.41)
Test for venereal diseases	62 (1.90)
Sexually transmitted diseases test	53 (1.64)
**Treatment (*n* = 985)**	
Clap treatment	101 (10.20)
Physician for venereal diseases	71 (7.25)
Physician venereal diseases	52 (5.25)
Venereal diseases physician	51 (5.17)
Venereal diseases which physician	40 (4.07)
**Transmission (*n* = 2464)**	
Chlamydia transmission	195 (7.91)
Venereal diseases oral	64 (2.59)
Clap incubation period	63 (2.54)
Clap transmission	62 (2.51)
Chlamydia infection without intercourse	55 (2.25)
**General (*n*= 4448)**	
Venereal diseases	2203 (49.52)
Sexually transmitted diseases	209 (4.7)
Venereal diseases pictures	65 (1.45)
Skin and venereal diseases	60 (1.35)
Venereal diseases list	56 (1.25)

^1^ Number of searches/100,000 inhabitants.

## Data Availability

The data can be obtained from the Corresponding Author upon request.

## Supplementary Materials

The following are available online at https://www.mdpi.com/1660-4601/18/5/2771/s1, Table S1: The five most-searched-for terms across all examined cities expressed as search queries per 100,000 inhabitants.
