# Supplementary material for: Interest in Sexually Transmitted Infections: Analysis of Web Search Data Terms in Eleven Large German Cities from 2015 to 2019
Source: Int J Environ Res Public Health. 2021 Mar 9;18(5):2771. doi: 10.3390/ijerph18052771 (PMC7975972; doi:10.3390/ijerph18052771)
Supplement: Supplementary file 1 [file ijerph-18-02771-s001.zip › SupplementaryTable1.docx]

**Supplementary Table 1.** The five most-searched-for terms across all examined cities expressed as search queries per 100,000 inhabitants.

| **Search terms** | **n^a^ (%)** |
| --- | --- |
| Chlamydia | 9,820 (23.71) |
| Genital warts | 4,595 (11.10) |
| HIV | 3,767 (9.10) |
| Clap | 3,088 (7.46) |
| Venereal diseases | 2,203 (5.32) |

^a^ Number of searches/100,000 inhabitants
